# Cytotoxic *Escherichia coli* strains encoding colibactin, cytotoxic necrotizing factor, and cytolethal distending toxin colonize laboratory common marmosets (*Callithrix jacchus*)

**DOI:** 10.1038/s41598-020-80000-1

**Published:** 2021-01-27

**Authors:** Colleen S. McCoy, Anthony J. Mannion, Yan Feng, Carolyn M. Madden, Stephen C. Artim, Gina G. Au, Mikayla Dolan, Jennifer L. Haupt, Monika A. Burns, Alexander Sheh, James G. Fox

**Affiliations:** 1 Division of Comparative Medicine, Massachusetts Institute of Technology, Building 16-825, 77 Massachusetts Avenue, Cambridge, MA 02139 USA; 2 Present Address: Merck Research Laboratories, Merck, South San Francisco, CA 94080 USA

**Keywords:** Animal disease models, Gastrointestinal models, Infectious diseases, Microbiology, Gastroenterology, Inflammatory bowel disease

## Abstract

Cyclomodulins are virulence factors that modulate cellular differentiation, apoptosis, and proliferation. These include colibactin (*pks*), cytotoxic necrotizing factor (*cnf*), and cytolethal distending toxin (*cdt*). Pathogenic *pks*+, *cnf*+, and *cdt*+ *E. coli* strains are associated with inflammatory bowel disease (IBD) and colorectal cancer in humans and animals. Captive marmosets are frequently afflicted with IBD-like disease, and its association with cyclomodulins is unknown. Cyclomodulin-encoding *E. coli* rectal isolates were characterized using PCR-based assays in healthy and clinically affected marmosets originating from three different captive sources. 139 *E. coli* isolates were cultured from 122 of 143 marmosets. The *pks* gene was detected in 56 isolates (40%), *cnf* in 47 isolates (34%), and *cdt* in 1 isolate (0.7%). The prevalences of *pks*+ and *cnf*+ *E. coli* isolates were significantly different between the three marmoset colonies. 98% of cyclomodulin-positive *E. coli* belonged to phylogenetic group B2. Representative isolates demonstrated cyclomodulin cytotoxicity, and serotyping and whole genome sequencing were consistent with pathogenic *E. coli* strains. However, the presence of *pks*+, *cnf*+, or *cdt*+ *E. coli* did not correlate with clinical gastrointestinal disease in marmosets. Cyclomodulin-encoding *E. coli* colonize laboratory common marmosets in a manner dependent on the source, potentially impacting reproducibility in marmoset models.

## Introduction

*Escherichia coli* is a ubiquitous and diverse species of Gram-negative, facultatively anaerobic bacilli in the family *Enterobacteriaceae*. Most strains of *E. coli* are commensals within the gastrointestinal tract of animals. However, pathogenic *E. coli* strains cause a variety of intestinal and extraintestinal infections ranging in severity from self-limiting to lethal^1^. These strains are classically categorized into pathotypes, including enteropathogenic *E. coli* (EPEC), uropathogenic *E. coli* (UPEC), meningitis-associated *E. coli* (MNEC), and others. Each pathotype represents a collection of strains with similar virulence factors conferring the ability to cause similar pathology^2^.

Cyclomodulins are a heterogeneous class of virulence factors that alter the eukaryotic cell cycle to promote bacterial invasion and host colonization. These actions induce genetic instability that may lead to the development of cancer. Cyclomodulins that are found in *E. coli* include colibactin, cytotoxic necrotizing factor (CNF), cytolethal distending toxin (CDT), cycle inhibiting factor, shiga toxin, and subtilase toxin^3^.

Colibactin is a genotoxic secondary metabolite produced by *Enterobacteriaceae* species harboring the polyketide synthase (*pks*) genomic island. Colibactin from *E. coli* alkylates adenine, causing DNA interstrand crosslinks and double strand breaks, leading to cell cycle arrest and senescence in vitro, as well as increased virulence in extraintestinal infections and tumor development in vivo^4–6^. The presence of *pks*+ *E. coli* is associated with inflammatory bowel disease (IBD) and colorectal cancer, and was recently shown to cause a unique mutational signature that is enriched within a subset of human cancers^7–9^. The highly reactive and unstable cyclopropane warheads of colibactin are activated during secretion to prevent autotoxicity to the bacteria; therefore, toxicity in vitro requires direct contact of cells with live bacteria. Effects on cells in vitro include megalocytosis, phosphorylated γ-H2AX foci, and G2 cell-cycle arrest^10,11^.

Cytotoxic necrotizing factor (CNF) is a family of AB toxins produced by UPEC strains that cause cytoskeletal changes resulting in macropinocytosis of bacteria into the host cell as well as G2 cell cycle arrest. These actions impair epithelial turnover and favor *E. coli* colonization. Consistent with these mechanisms, many cell lines treated with sonicates of *cnf*+ *E. coli* exhibit multinucleation and ruffled cell borders. CNF1 can also induce epithelial to mesenchymal transition in vitro, and thus may increase the risk of cancer^12^. Three CNF types are described in *E. coli*, however, CNF2 and CNF3 are not frequently detected^13,14^.

UPEC strains also produce hemolysin, a membrane pore-forming toxin. The *cnf1* gene, when present, is always encoded on the *hlyCABD* operon and is co-transcribed with hemolysin. However, *hlyCABD* can exist without *cnf1*^15^. Hemolysin is produced as either a free form or an outer membrane vesicle (OMV)-associated form. The free form irreversibly inserts into cell membranes of erythrocytes and epithelial cells, where depending on concentration it causes ion imbalance, structural changes, and cell lysis. The OMV-associated form is internalized by epithelial cells, where it targets mitochondria and triggers caspase-9-mediated apoptosis^16–18^.

Cytolethal distending toxin (CDT) is an AB_2_-type toxin produced by several pathogenic Gram-negative bacteria which causes DNA damage and cell cycle arrest. The catalytic subunit CdtB is highly conserved between species and causes single and double-strand DNA breaks via DNase I-like activity. This triggers the DNA damage response via ATM kinase, leading to both G2/M and G1/S cell cycle arrest. CDT increases bacterial gut colonization, promotes pro-inflammatory responses, and dysregulates the immune response. Treatment of HeLa cells with CDT in vitro causes cellular distension and multinucleation^3,19^.

The common marmoset (*Callithrix jacchus*) is a nonhuman primate species that is increasingly used in a variety of biomedical research fields including neuroscience, toxicology, infectious disease, immunology, reproduction, obesity, and aging^20^. Common marmosets in captivity are frequently afflicted with IBD-like disease, which is often diagnosed post-mortem as chronic lymphocytic enteritis (CLE)^21^, the etiology and pathogenesis of which are presently unknown. The potential role of cyclomodulin-encoding enteropathogenic *E. coli* in the pathogenesis of IBD-like disease of marmosets has not previously been investigated, although *E. coli* has previously been associated with GI disease in marmosets^22–24^.

Most captive marmoset colonies are not specific pathogen free (SPF), and none exclude *E. coli*. As such, the prevalence of cyclomodulin-encoding *E. coli* in laboratory marmosets is currently unknown. We hypothesized that common marmosets, similar to laboratory rodents and other laboratory nonhuman primates^25–29^, are colonized by cyclomodulin-encoding *E. coli*, and the prevalence of *pks*+, *cnf*+, and *cdt*+ *E. coli* in our population would vary significantly depending on the colony of origin, as has been demonstrated in laboratory rats^28^. In addition, we hypothesized that *pks*+, *cnf*+, and *cdt*+ *E. coli* are present in animals afflicted with gastrointestinal disease with greater frequency than in clinically normal animals. Additionally, colonization of marmosets by cyclomodulin-encoding *E. coli* might be expected to induce physiological variability between animals in a study, potentially impacting reproducibility.

## Results

### Microbiologic characterization of marmoset *E. coli* isolates

In total, 139 *E. coli* strains were isolated from 122 of the 143 marmosets sampled in all three colonies (Supplementary Table S1). In Colony A, 31 strains of *E. coli* were isolated from 26 of the 33 animals. In Colony B, 33 strains of *E. coli* were isolated from 32 of the 33 animals. In Colony C, 75 strains of *E. coli* were isolated from 64 of the 77 animals. Eight *E. coli* isolates originating from Colony C demonstrated hemolytic activity when cultured on blood agar plates. Some animals harbored multiple *E. coli* isolates as determined by distinct colony morphology and biochemical characterization by API testing. Of the 139 total *E. coli* isolates, 54 were API code 5144572, and 51 isolates were API code 5144552, constituting the majority of isolates present in all three marmoset colonies. The major metabolic difference between these codes is that strains with API code 5144572 ferment sucrose, whereas those with 5144552 did not. Other API codes represented were 5044552, 5144172, 7144552, 7145552, 5044572, 5144573, 1044552, 4144512, and 7144572 (Supplementary Figure S1). There were significant associations between marmoset colony of origin and *E. coli* strain-linked biochemical characteristics, including the presence of ornithine decarboxylase (p < 0.001), butylene glycol pathway (p = 0.01), sucrose fermentation (p < 0.001), and amygdalin fermentation (p < 0.05) (Supplementary Figure S1).

### Phylogenetic distribution of marmoset *E. coli* isolates

**Figure 1:**
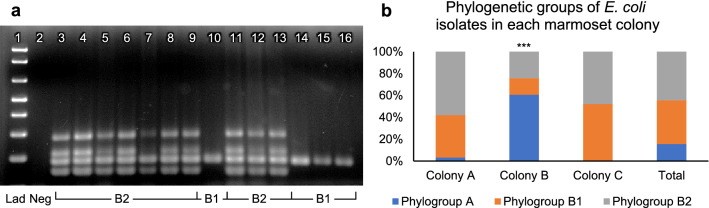
Phylogenetic analysis of *E. coli* isolates from marmosets. (**a**) Sets of primers for *svg*, *chuA, yjaA*, *uidA*, and TspE4.C2 genes were used in multiplex PCR assays to determine the phylogroup of each isolate. The phylogenetic groups were determined according to the PCR gel pattern, with the presence of 3 or more bands indicating membership in phylogroup B2. Phylogroup multiplex PCR gel: lane 1,1-kb ladder; lane 2, negative control; lane 3, phylogroup B2 positive control (NC101); lanes 4–16, *E. coli* isolates from marmosets (lanes 3–9 and 11–13, phylogroup B2; lanes 10 and 14–16, phylogroup B1). (**b**) Distribution of phylogenetic groups of *E. coli* isolated from marmosets according to colony. Colony B had a significantly different distribution than either of the other two colonies. *** p < 0.001, Fisher’s Exact Test. Unedited images of gels are shown in Supplementary Figure S6.

Phylogenetic group was determined according to the specific gel electrophoresis banding pattern resulting from multiplex PCR amplification of genes *svg*, *chuA, yjaA*, *uidA*, and TspE4.C2 (Fig. 1a). Isolates in phylogroup A were positive for *yjaA* and *uidA*, phylogroup B1 were positive for *uidA*, and phylogroup B2 were positive for *chuA*, *yjaA*, *uidA*, and TspE4.C2. Out of 135 total isolates tested, 21 were in phylogroup A (16%), 54 were in phylogroup B1 (40%), and 60 were in phylogroup B2 (44%) (Fig. 1b). In marmoset colony A, 1 of the 31 isolates was phylogroup A, 12 were phylogroup B1, and 18 were phylogroup B2. In marmoset colony B, 20 of the 33 isolates were in phylogroup A, 5 were in phylogroup B1, and 8 were in phylogroup B2. In marmoset colony C, 37 of the 71 isolates were in phylogroup B1, 34 were in phylogroup B2, whereas none of the isolates in marmoset colony C were in phylogroup A. The distribution of *E. coli* phylogroups was significantly different in marmoset colony B compared to *E. coli* isolates from the other two colonies (p < 0.001). Phylogenetic group correlated strongly with genotype, in that all phylogroup A isolates, and all but one of the phylogroup B1 isolates, were negative for *pks*, *cnf*, and *hlyA*. Of the 60 phylogroup B2 isolates, 54 were cyclomodulin-positive. All 6 phylogroup B2 isolates that were *pks*-/*cnf*- were cultured from marmoset colony B.

### Distribution of marmoset *E. coli* isolates encoding cyclomodulins

**Figure 2:**
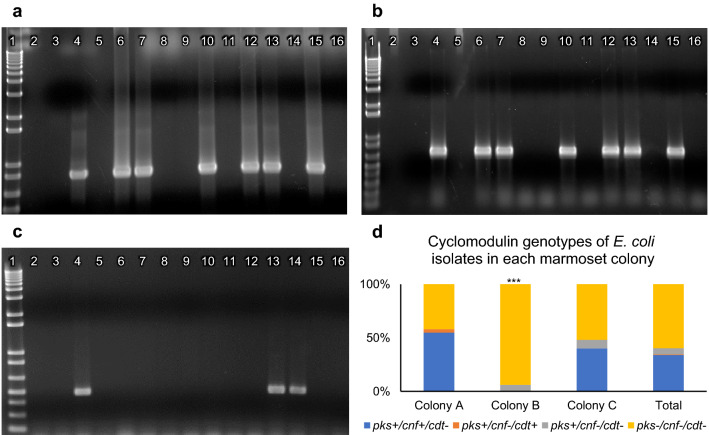
PCR amplification of cyclomodulin genes in representative *E. coli* isolates from marmosets. (**a**) Amplification of *clbQ* gene from *pks* island. (**b**) Multiplex PCR amplification was used to detect both chromosomal and plasmid *cnf* genes. (**c**) Multiplex PCR amplification was used to detect all known variants of the *cdtB* gene. PCR gels: lane 1,1-kb ladder; lane 2, blank; lane 3, negative control; lane 4, positive control; lanes 5 through 16, representative *E. coli* isolates from marmosets. Both *cdt*-positive isolates shown (lanes 13 and 14) are from the same animal. (**d**) Distribution of cyclomodulin genotypes of *E. coli* isolated from marmosets according to colony. Colony B had a significantly different distribution than either of the other two colonies. *** p < 0.001, Fisher’s Exact Test. Unedited images of gels are shown in Supplementary Figure S6.

All *E. coli* isolates were tested by PCR for cyclomodulin genes with primers for the *clbQ* gene of the *pks* island (Fig. 2a), with multiplex primers to amplify both chromosomal and plasmid *cnf* genes (Fig. 2b), and with multiplex primers to amplify all known variants of *cdtB* genes (Fig. 2c). Of the 139 *E. coli* isolates, 56 were positive for *pks* (40.3%), 47 were positive for *cnf* (33.8%), and 1 was positive for *cdt* (0.7%) (Supplementary Table S2). All 47 *cnf*+ isolates were also *pks*+. Of the 9 *pks*+/*cnf*- isolates, one was *cdt*+. (Fig. 2d) The single animal with *cdt*+ *E. coli* had a similar *pks*+/*cnf*−/*cdt*+ *E. coli* isolate simultaneously cultured from a nasal swab taken as a diagnostic sample, and the PCR bands from both isolates are depicted in Fig. 2c. The *cdtB* amplicon sequences of both *E. coli* isolates from this animal aligned with 98% identity to the gene for CDT Type IV subunit B found in *E. coli* strain NCTC 8196. The remaining 83 isolates were negative for all three cyclomodulin genes tested. From marmoset colony A, 17 of the 31 *E. coli* isolates were *pks*+/*cnf*+/*cdt*−, one isolate was *pks*+/*cnf*−/*cdt*+, and the remaining 13 isolates were *pks*-/*cnf*-/*cdt*-. In marmoset colony B, 2 of the 33 *E. coli* isolates were *pks*+, and all of the 33 isolates were *cnf*-. In marmoset colony C, 36 of the 75 *E. coli* isolates were *pks*+, and 6 of those were also *cnf*-. The remaining 39 *E. coli* isolates from marmoset colony C were *pks*-/*cnf*-. The prevalence of both *pks* and *cnf* were significantly lower in colony B compared to the other two marmoset colonies (p < 0.001).

Captive marmosets are maintained in cohoused family units, which may influence transmission of *E. coli* within colonies. Therefore, we evaluated if *E. coli* strains differed between family lines in each colony. The two largest extended family lines (Family 1 and Family 2) in Colony C consisted of cohoused parents, adult offspring that were previously cohoused with the parents, and unrelated mates cohoused with the offspring. These two families were found to have a significantly higher proportion of *pks*+/*cnf*− *E. coli* isolates (24% prevalence) than the rest of Colony C (0% prevalence) (p < 0.001) (Supplementary Figure S2). Similar *pks*+/*cnf*− *E. coli* isolates had been cultured from these animals over the previous two years (Supplementary Figure S2). The cyclomodulin prevalence in *E. coli* isolates from other large families were not significantly different from *E. coli* isolates in their colonies of origin.

To determine if marmosets are stably colonized over time with cyclomodulin-encoding *E. coli*, we analyzed data from animals which had been sampled multiple times over the course of two years. Interestingly, of the 51 animals that had been sampled 12 and 24 months previously, 5 consistently had *pks*+/*cnf*+/*cdt*− *E. coli*, 6 consistently had *pks*+/*cnf*−/*cdt*− *E. coli*, and an additional 20 marmosets consistently had *pks*−/*cnf*−/*cdt*− *E. coli* isolated from rectal swabs (Supplementary Figure S2).

### Identification of mutant hemolysin gene

Cytotoxic necrotizing factor, when present, is always encoded on the same operon with hemolysin, though it is common for an isolate to encode hemolysin without CNF^15^. Therefore, it was surprising when 45 of the 47 *cnf*+ *E. coli* isolates did not demonstrate hemolytic activity when cultured on blood agar. The presence of hemolysin was tested by PCR in 136 *E. coli* isolates by amplification of the gene *hlyA* (Supplementary Figure S3). The *hlyA* PCR product size was 584 bp, which was produced from all eight hemolytic isolates. The sequence of this amplicon demonstrated 100% identity with the hemolysin A gene from 16 different *E. coli* reference isolates, including UTI89. Forty-two out of 136 marmoset *E. coli* isolates produced a 1665 bp amplicon, all of which were non-hemolytic and *cnf*+ . The *hlyA* gene in these longer amplicons was disrupted by an IS481-like element ISKpn28 family transposase, an insertional sequence previously found in *Klebsiella pneumoniae* isolates^30^. The mutant hemolysin was thus designated *hlyA::ISKpn28*. All three marmoset colonies had significantly different prevalence of wildtype and mutant hemolysin genes, with Colony A having proportionally more *E. coli* isolates with *hlyA::ISKpn28*, all Colony B isolates being negative for both wildtype and mutant hemolysin, and all wildtype *hlyA*+ isolates originating from Colony C (p < 0.01) (Supplementary Figure S3).

### Clinical correlates

**Figure 3:**
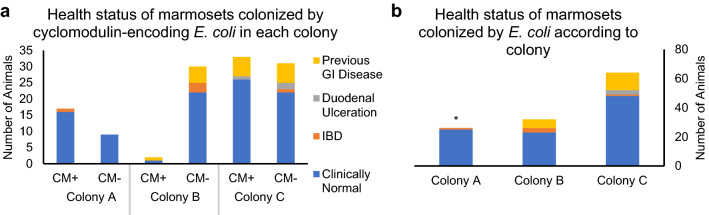
(**a**) Health status of captive marmosets was not significantly different between animals that were or were not colonized by cyclomodulin-encoding *E. coli* isolates (as determined by PCR for *pks*, *cnf*, or *cdt*), nor was there a significant colony effect. (**b**) Health status of captive marmosets was significantly different in Colony A from the other two colonies. * p < 0.05, Fisher’s Exact Test.

Medical records were evaluated for temporal correlations of GI clinical signs from animals which had cultured *pks*+, *cnf*+, or *cdt*+ *E. coli* isolates. Of 122 animals from which *E. coli* was cultured, 96 had no history of GI disease, 18 had a history of clinical GI disease but were asymptomatic at the time of sample collection, 5 had IBD-like disease, and 3 were previously diagnosed with duodenal ulcers. There were no significant associations of gastrointestinal disease with the presence or absence of cyclomodulin-encoding *E. coli* overall, or when sorted by colony (Fig. 3).

### In vitro cytotoxicity of *E. coli* isolates from marmosets

Cell culture assays were performed to demonstrate cytotoxicity of nine selected representative *E. coli* isolates (S1-S9) to HeLa cells. To detect colibactin activity, cells must be exposed to live bacteria to allow for precolibactin transport into the bacterial periplasmic space, followed by maturation^31^. Conversely, CDT and CNF cytotoxicity are only detectable using sonicate or supernatant preparations^32^. Live *pks*+ *E. coli* isolates S1-S6 induced megalocytic cytotoxicity in HeLa cells in a manner corresponding to multiplicity of infection, indicating contact-dependent colibactin activity (Supplementary Figure S4). HeLa cells treated with sonicate from *cnf*+ *E. coli* isolates S1-S3 displayed multinucleation and megalocytic cytotoxicity in a dose-dependent manner (Supplementary Figure S4). HeLa cells treated with sonicate from the *cdt*+ *E. coli* isolate S6 displayed megalocytosis and multinucleation in a dose-dependent manner (Supplementary Figure S4). These phenotypes were not observed in HeLa cells treated with live *pks*- *E. coli* isolates S7, S8, and S9, or with the sonicates of *cnf*− or *cdt*− isolates S4, S5, S7, S8, and S9.

### Serotyping

Nine representative *E. coli* isolates (S1-S9) from all three colonies were sent to the *E. coli* Reference Center at Pennsylvania State University for serotyping and further virulence factor profiling (Table 1). Three of the isolates were O6:H+, all of which were in phylogenetic group B2 and *pks*+, but otherwise differed by genotype and source colony. Two of the isolates were O2:H14, both of which were in phylogenetic group B2 and encoded *pks*, *cnf*, and the mutant hemolysin gene, but each isolate originated from a different colony. The remaining *pks*+ isolate was *cdt*+, serotype O7:H7, and was in phylogenetic group B2. The three remaining isolates represented all three marmoset colonies and were negative for all tested virulence factors. The isolate from marmoset colony A was serotype O128:H2 and phylogroup B1. The isolate from marmoset colony B was serotype O26:H32 and phylogenetic group B2, which was unusual for a *pks*- *E. coli* isolate. The isolate from marmoset colony C was serotype O139:H19 and phylogroup B1. This isolate tested positive for *cnf1* by PCR at the *E. coli* Reference Center at Pennsylvania State University, which conflicted with our negative PCR result as well as with later genome sequencing results. None of the nine *E. coli* isolates serotyped were positive for *elt*, *estA*, *estB*, *stx1*, *stx2*, *eae*, or *cnf2*.

**Table 1:** Serotype and virulence factor profiling results of representative *E. coli* isolates from marmosets. Assays were conducted by *E. coli* Reference Center at The Pennsylvania State University. S8 had conflicting results on *cnf1* between our primers and Penn State’s primers. *elt*, heat-labile enterotoxin; *estA*, heat-stable enterotoxin A; *estB*, heat-stable enterotoxin B; *stx1*, Shiga-type toxin 1; *stx2*, Shiga-type toxin 2; *cnf1*, cytotoxic necrotizing factor 1; *cnf2*, cytotoxic necrotizing factor 2; *eae*, intimin gamma.

Strain	Marmoset colony	Cyclomodulin genotype	*hlyA*	Phylo-group	Serotype	*elt*	*estA*	*estB*	*stx1*	*stx2*	*eae*	*cnf1*	*cnf2*
S1	C	*pks* + /*cnf* + /*cdt*-	Mutant	B2	O2:H14	−	−	−	−	−	−	+	−
S2	A	*pks* + /*cnf* + /*cdt*-	Mutant	B2	O2:H14	−	−	−	−	−	−	+	−
S3	C	*pks* + /*cnf* + /*cdt*-	+	B2	O6:H +	−	−	−	−	−	−	+	−
S4	C	*pks* + /*cnf*-/*cdt*-	+	B2	O6:H +	−	−	−	−	−	−	−	−
S5	B	*pks* + /*cnf*-/*cdt*-	-	B2	O6:H +	−	−	−	−	−	−	−	−
S6	A	*pks* + /*cnf*-/*cdt* +	-	B2	O7:H7	−	−	−	−	−	−	−	−
S7	B	*pks*-/*cnf*-/*cdt*-	-	B2	O26:H32	−	−	−	−	−	−	−	−
S8	C	*pks*-/*cnf*-/*cdt*-	-	B1	O139:H19	−	−	−	−	−	−	+	−
S9	A	*pks*-/*cnf*-/*cdt*-	-	B1	O128:H2	−	−	−	−	−	−	−	−

### Draft genome sequencing and comparative analysis

Whole genome sequences of five representative *E. coli* isolates (S1, S3, S4, S5, and S8) were evaluated for the presence of known virulence factor genes and for comparative analysis with other cyclomodulin-encoding *E. coli* from humans, rhesus macaques, and laboratory rodents. The genome sizes, GC contents, protein-coding sequences and RNA genes of the representative marmoset *E. coli* isolates were comparable to those of *pks*+ *E. coli* strains IHE3034 and NC101, as well as *cnf*+ *E. coli* strain UTI89 (Table 2). Gene sequences homologous to the *pks* island found in IHE3034 and NC101 were present in marmoset *E. coli* isolates S1, S3, S4, and S5, as predicted by the results of PCR and in vitro cytotoxicity assays. Syntenic alignments were confirmed for the *pks* island in all four predicted marmoset *E. coli* isolates with *E. coli* strains IHE3034 and NC101. (Supplementary Figure S5) Isolate S4 was found to have a putative hybrid gene for *clbJ-K*, similar to a gene sequence found previously in *E. coli* isolates from laboratory and pet rats^28,33^.

**Table 2:** Novel marmoset *E. coli* genomes have similar statistics as pathogenic, *pks*-encoding *E. coli* strains IHE3034 and NC101, and *cnf*-encoding uropathogenic *E. coli* strain UTI89. Virulence factor genes for toxins, survival factors, and adhesins, as well as antibiotic resistance genes, were identified in the marmoset *E. coli* genomes.

Strain	Isolation source	Genome length (bp)	Contigs	G+C% content	Protein-coding sequences	tRNA/rRNA	Virulence factor and antibiotic resistance genes*	GenBank accession
S1	Research marmoset	5,135,378	98	50.62	5074	77/10	*pks, cnf1, pic, vat, gad, iroN, cba, cma, mcmA, mchB, mchC, mchF, mdfA*	JAADCB000000000
S3	Research marmoset	5,136,704	91	50.50	5133	78/11	*pks, cnf1, pic, vat, iss, iroN, sfaS, mcmA, mdfA*	JAADBZ000000000
S4	Research marmoset	5,190,399	112	50.52	5252	86/10	*pks, pic, vat, iss, iroN, sfaS, mcmA, mdfA*	JAADCA000000000
S5	Research marmoset	5,168,141	129	50.40	5203	78/10	*pks, vat, iss, iroN, sfaS, mcmA, mdfA*	JAADCC000000000
S8	Research marmoset	4,590,816	64	50.78	4442	79/9	*gad, lpfA, mdfA*	JAADBY000000000
IHE3034	Human neonatal meningitis	5,108,383	complete genome	50.70	5045	97/22	*pks, vat, gad, iss, iroN, sfaS, mdfA, cdtABC*	CP001969.1
NC101	Research mouse	5,021,144	27	50.57	4917	72/4	*pks, vat, gad, iss, iroN, sfaS*	AEFA00000000.1
UTI89	Human uropathogenic	5,179,971	complete genome	50.60	5040	89/14	*cnf1, vat, gad, iss, iroN, sfaS, senB*	CP000243.1
K12 substrain DH10B	Human nonpathogenic	4,686,137	complete genome	50.80	4606	87/14	*gad, iss, mdfA*	CP000948.1

* Gene names: *pks*, polyketide synthetase (colibactin); *cnf1*, cytotoxic necrotizing factor; *pic*, serine protease autotransporters of *Enterobacteriaceae* (SPATEs); *vat*, vacuolating autotransporter toxin; *gad*, glutamate decarboxylase; *iss*, increased serum survival; *iroN*, enterobactin siderophore receptor protein; *lpfA*, long polar fimbriae; *sfaS*, S-fimbriae minor subunit; *cba*, colicin B; *cma*, colicin M; *mcmA*, microcin M; *mchB*, microcin H47 biosynthesis protein; *mchC*, microcin H47 biosynthesis protein; *mchF*, ATP-binding cassette transporter protein for microcin H47; *mdfA*, multidrug efflux pump; *cdtABC*, cytolethal distending toxin subunits A, B, and C; *senB*, enterotoxin TieB protein.

Gene sequences homologous to the *hlyCABD*-*cnf1* operon present in UTI89 were detected in marmoset *E. coli* isolates S1 and S3, as predicted by the results of PCR and in vitro cytotoxicity assays. Syntenic alignments were confirmed for the *hlyCABD*-*cnf1* operon in all predicted marmoset *hlyA*+ *E. coli* isolates with *E. coli* strain UTI89 (Supplementary Figure S5). Isolate S1 contained a 1081 bp insertion in the *hlyA* gene sequence as predicted by PCR and amplicon sequencing. Isolate S4 encoded all four hemolysin genes, but not the *cnf1* gene sequence, consistent with PCR results, in vitro cytotoxicity in HeLa cells, and hemolytic phenotype.

Additional virulence factor genes were detected in the marmoset *E. coli* isolates (Table 2), including serine protease autotransporters of *Enterobacteriaceae* (SPATEs) (*pic*, *vat*), survival and immune evasion factors (*gad*, *iss*), iron acquisition (*iroN*), adherence (*lpfA, sfaS*), and synthesis and secretion genes for bacteriocins (*cba*, *cma, mcmA*, *mchB*, *mchC*, *mchF*). All five marmoset *E. coli* isolates encoded the *mdfA* gene, a broad-spectrum multidrug efflux pump that contributes to antibiotic resistance. Sequences for other cyclomodulins *cnf2*, *cdtABC* and *cif*, were not detected. Genes for virulence factors *elt*, *estA*, *estB*, *stx1*, *stx2*, and *eae* were not detected, consistent with previous PCR results. Several isolates (S1, S3, S4, and S5) had similar virulence factor profiles to necrotoxigenic (NTEC) and uropathogenic (UPEC) strains of *E. coli*, including CNF1, α-Hemolysin (αHly), fimbrial adhesins (SfaS), increased serum survival (ISS), and siderophore receptors (IroN). Similar Genome Finder showed isolates S3, S4 and S5 closely matched UPEC strains that caused urinary tract infections and bacteremia in humans and animals worldwide. Isolate S1 was similar to antibiotic resistant strains from human feces, and S8 showed similarity to potential foodborne contaminants. Similar genome descriptions and references are listed in Supplementary Table S3.

## Discussion

*Escherichia coli* is normally a commensal organism colonizing the lower GI tract of common marmosets. However, certain strains of *E. coli* encode virulence factors which can cause disease. Enteropathogenic *E. coli* (EPEC) has previously been implicated in diarrhea, hemorrhagic typhlocolitis, and ileitis in laboratory marmosets^22–24^. Marmoset EPEC strains demonstrated similarities in virulence factors, pathogenic properties, and genomic features to human strains, indicating they are likely equivalent pathotypes and possibly capable of zoonotic transmission^22,34^. Additionally, marmosets have been experimentally infected with uropathogenic *E. coli* (UPEC) which caused lower urinary tract infection and pyelonephritis^35^. However, the prevalence and pathogenicity of cyclomodulin-encoding *E. coli* in laboratory common marmosets is not currently known.

Because of the diversity and ubiquity of *E. coli*, it can be challenging to determine whether an isolated strain is pathogenic. This determination is made based on the source, biotype, serotype, phylogenetic background, virulence factor profile, and demonstration of virulence properties using in vitro and in vivo models. Biotyping is commonly used to phenotypically differentiate unique *E. coli* isolates by characterization of the biochemical metabolism profile. Many marmosets were shown to be colonized by multiple unique *E. coli* isolates by this method. Phylogenetic typing is a simple method using multiplex PCR to assign an isolate into one of five phylogroups, which significantly correspond with the isolate’s pathogenicity^36^. This makes it feasible for use on a large scale to screen for potentially pathogenic *E. coli* in a given population. Pathogenic *E. coli* strains typically belong to phylogroups B2 or D, whereas those in phylogroups A and B1 are usually considered commensals^37^. Ninety-eight percent of the cyclomodulin-encoding *E. coli* isolated from laboratory marmosets belong to phylogenetic group B2, which is consistent with strains isolated from humans and other species^11,26–28,33,38^. The distribution of phylogroups was significantly different in Colony B, which had 61% prevalence of the non-pathogenic phylogroup A, whereas the prevalence of phylogroup A in Colony A was only 3%, and it was not isolated in Colony C. Potentially pathogenic *E. coli* classified in phylogroup B2 had a 44% prevalence overall, and was associated with the presence of virulence factor genes *pks*, *cnf* and *hlyA*, especially in Colonies A and C. Marmoset colony B had a significantly lower prevalence of *pks*+ (6%) and *cnf*+ (0%) *E. coli* isolates than the other two colonies.

Overall, 40% of the laboratory marmoset *E. coli* isolates were *pks*+. This is similar to the prevalence of *pks*+ *E. coli* in laboratory macaques (30.1%)^26^. Prevalence of *pks*+ *E. coli* in healthy humans ranged from 4.3 to 22%^7,39^. The majority (84%) of *pks*+ *E. coli* isolates from marmosets were also *cnf*+, and all *cnf*+ *E. coli* isolates were also *pks*+. Double-positive isolates (*pks*+/*cnf*+) have been characterized from both healthy humans and patients with urosepsis^38^, in contrast to surveys in humans and macaques where *cnf* is occasionally present in *pks*− isolates^26,40^. Nearly 34% of the marmoset *E. coli* isolates were *cnf*+, similar to what has been found previously in other laboratory nonhuman primates (20.9%)^26^. In contrast, a similar survey of *cnf*+ *E. coli* in healthy humans showed a prevalence of only 2%^38^.

Only one *E. coli* isolate was *cdt*+ (0.7% prevalence). Other surveys have not found *cdt*+ *E. coli* in human patients with diverticulosis, nor in macaques^26,32^.

Previous studies have shown an association between *cnf* and hemolysis^29,38,40^, which is consistent with the proximity of the *cnf* gene to the hemolysin gene. Interestingly, 96% of the *cnf* + isolates from marmosets did not demonstrate hemolysis due to an insertion event in the *hlyA* gene. Strains of *E. coli* with a similar deactivating insertion in the *hlyA* gene were previously isolated from laboratory rats from a specific vendor^28^.

HeLa cells infected with live bacteria of select *pks*+ *E. coli* isolates demonstrated megalocytosis, cytopathic morphology consistent with colibactin, and depending on the multiplicity of infection (MOI). In this assay, the cytopathic effect requires direct contact with live cells, presumably due to the instability of colibactin, which is matured upon secretion. Consistent with this known mechanism, HeLa cells treated with sonicates of *pks*+/*cnf*−/*cdt*− *E. coli* were indistinguishable from those treated with nonpathogenic K12 *E. coli*. HeLa cells treated with sonicates of select *cnf*+ *E. coli* isolates demonstrated multinucleation and megalocytosis, morphology consistent with cytotoxicity from CNF, in a manner dependent on the protein concentration of *cnf*+ *E. coli* sonicates. HeLa cells treated with the sonicate from S6, the single *cdt*+ *E. coli* isolate demonstrated cellular distension and multinucleation consistent with cytotoxicity from CDT and dependent on the protein concentration of the sonicate. Cells treated with cyclomodulin-negative *E. coli* strains were indistinguishable from those treated with nonpathogenic K12 strain. These results were consistent with findings from previous studies showing cytotoxic effects of *pks*+, *cnf*+ and *cdt*+ *E. coli *in vitro^11,26,28,29,33^.

Serotyping uses antibodies to characterize the O-polysaccharide antigens, flagellar H-antigens, and capsular K-antigens found on the surface of the bacteria. Certain serotypes are more frequently associated with specific pathotypes, such as EHEC O157:H7^41^. Serotypes of select *E. coli* isolates from marmosets suggested the potential for pathogenicity. The most common serotypes were O2 (S1–S2) and O6 (S3–S5) (Table 1), both of which are frequently associated with urinary tract infection (UTI) in humans and animals^42,43^. Genome sequence analysis supports the potential of these isolates to cause UTI. Virulence factors associated with UPEC pathotype were found in isolates S1–S5, including S-fimbriae (*sfaS*), hemolysin (*hlyA*), *cnf*, iron uptake genes (*iroN*), and other colonization and survival factors (*pic*, *vat*)^2^. Whether these *E. coli* serotypes are associated with UTI in marmosets requires further study. *E. coli* in serogroups O26 and O128:H2 have been associated with enterohemorrhagic *E. coli* (EHEC) infections in humans and animals^44^. However, these isolates (S7 and S9) tested negative by PCR for the EHEC virulence factors *stx1*, *stx2*, and *eae*, in addition to being negative for *hlyA*, *pks*, and *cnf*, so they are unlikely to be pathogenic. Nonpathogenic O7:H7 *E. coli* negative for both *eae* and *stx* were isolated from laboratory rabbit fecal samples^45^. Finally, O139 is commonly involved in edema disease in post-weaning pigs^46^.

Whole genome sequence analysis of select *E. coli* isolates raised an interesting question about genetic variants of *pks*. Isolate S4 had a putative hybrid gene for *clbJ-K*. This hybrid gene contains approximately 90% of the *clbJ* sequence, and 45% of the *clbK* sequence, when compared to the IHE3034 genome. The putative *clbJ-K* hybrid gene is predicted to translate into a 2440-amino acid hybridized protein that contains two NPRS modules as well as an oxidase domain. Isolate S4 exhibited cytotoxicity to HeLa cells similar to other *pks*+ *E. coli* isolates tested (Supplementary Figure S4), suggesting the hybrid gene produces functional colibactin. Similar *clbJ-K* hybrid genes were found in *E. coli* isolates from laboratory and pet rats; and these isolates caused cytotoxicity and DNA damage in HeLa cells following in vitro infection^28,33^.

The fact that differences in prevalence were detected years after animals were transferred from each colony of origin to MIT suggests that these *E. coli* strains can stably colonize marmosets over multiple years. This is supported by the fact that similar *E. coli* isolates were cultured from 60% of marmosets 12 and 24 months previously. Furthermore, the presence of similar *E. coli* isolates in animals that were born at the MIT facility suggests these strains are passed to infants from family members. This observation is further validated by the unique cyclomodulin gene distribution found in Families 1 and 2 from Colony C, where 24% of individuals carried *pks*+/*cnf*− isolates of *E. coli* compared to 0% of the remainder of marmosets in Colony C.

Interestingly, 42/56 *pks*+ *E. coli* isolates harbored a hemolysin A gene with an inactivating insertional mutation (designated *hlyA::ISKpn28*). Thirty-four of the 42 *hlyA::ISKpn28* strains had identical biochemical profiles (API code 5144552), and two representative strains were serotype O2:H14. The remaining 8 *hlyA::ISKpn28* strains had identical biochemical profiles to each other (API code 7144552), and possessed arginine dihydrolase activity unlike the 34 *hlyA::ISKpn28* isolates. All 42 of these isolates were only cultured from Colony A and C animals, suggesting there could be clonal transmission within and between the marmoset colonies. Future studies using whole genome sequencing will be required to confirm clonality as well as the need to evaluate the dynamics of *E. coli* transmission in marmoset colonies and family units.

Common marmosets in captivity are frequently afflicted with IBD-like disease in which the etiology and pathogenesis are presently unknown. In humans, cyclomodulin-encoding *E. coli* have been demonstrated in higher frequency in patients with colorectal cancer, familial adenomatous polyposis, and IBD than in healthy controls or in patients with other GI diseases^7,9,32,39^. Although this study did not show an association between cyclomodulin-encoding *E. coli* and clinical GI disease in marmosets, it does not exclude their potential role in subclinical intestinal inflammation in marmosets. One limitation of this study is that the evolving nature of the clinical diagnosis of CLE in marmosets means that some of the animals in this study may have been in early subclinical stages of the disease. It is possible that some strains of *E. coli* present in the GI tract may not have been isolated from a rectal swab culture, although microbiome comparison of rectal swabs to feces in marmosets showed good agreement^47^. Finally, the fact that *pks* is present in other *Enterobacteriaceae* species raises the possibility that colibactin-producing bacteria are more prevalent in this population than we detected by culture of fecal material for *E. coli* only. These issues can be addressed in future studies with improved antemortem diagnostics including markers of intestinal inflammation, as well as clinical correlations with metagenomic data.

This report is the first to characterize the presence of cyclomodulin-encoding *E. coli* in marmosets. We have demonstrated that laboratory common marmosets can be colonized by cyclomodulin-encoding *E. coli* and have further shown that the prevalence of these cyclomodulin genes varies significantly depending on the source of the animal. Although we hypothesized that *pks*+, *cnf*+ and *cdt*+ *E. coli* is present in animals clinically afflicted with gastrointestinal disease at greater frequency than in clinically normal animals, no such association was detected in this cohort of marmosets. However, *E. coli* encoding colibactin, CNF, and to a lesser extent CDT, colonizing laboratory common marmosets may influence clinical and subclinical disease or contribute to physiological variation between animals from different sources, thus potentially affecting reproducibility in this model.

## Materials and methods

### Animals

Common marmosets (*Callithrix jacchus*) were housed in an AAALAC-accredited institution. Studies were conducted on an animal use protocol approved by the Massachusetts Institute of Technology Committee on Animal Care, and all experiments were performed in accordance with relevant guidelines and regulations. Marmosets were socially housed in breeding pairs or small family groups unless a suitable mate was unavailable. Enclosures were enriched stainless steel and polycarbonate cages (inner dimensions 56 in. × 28 in. × 28 in.) in a housing room maintained at 23.3 ± 1.0 °C, with a relative humidity of 30% to 70% and a 12:12-h light:dark cycle. Diet consisted of extruded biscuits (Teklad New World Primate Diet 8794, Envigo, Madison, WI) soaked lightly in water, supplemented with canned diet (ZuPreem, Premium Nutritional Products, Shawnee, KS), washed fruits and vegetables, and various protein sources. Chlorinated reverse-osmosis-purified water was provided ad libitum. Marmosets were observed at least twice daily by veterinary staff, and individual health records were maintained.

Marmosets originating from three different institutions were maintained in separate breeding colonies with strict biosecurity to minimize microbial contamination. Colony A came from a contract research organization in 2017. Colony B came from a commercial vendor in 2016. Colony C came from the New England Primate Center in 2014. Animals were seronegative for squirrel monkey cytomegalovirus, *Saimiriine herpesvirus 1*, *Saimiriine herpesvirus 2*, and measles virus (VRL Laboratories, San Antonio, TX) on arrival to MIT. Semiannual health monitoring included sedated physical examinations, hematology, and surveillance for fecal pathogens.

### Culture and isolation

Rectal swabs were collected during semiannual examinations from all marmosets over 6 months of age in 2018, and from a subset of animals in 2016 and 2017. Rectal swabs were enriched in Gram-negative broth (Becton Dickenson, Franklin Lakes, NJ), then streaked onto MacConkey lactose agar plates (Remel, Lenexa, KS). Lactose-fermenting colonies were selected, then plated onto sheep blood agar plates (Remel) to determine hemolysis. All bacterial cultures were incubated overnight at 37 °C. Isolates phenotypically consistent with *E. coli* were biochemically characterized using API 20 E (Biomérieux, Marcy l’Etoile, France).

### DNA extraction and PCR amplification

*Escherichia coli* colonies were suspended in sterile phosphate buffered saline (PBS), boiled for 10 min, and centrifuged at 12,000*g* for 10 min. Supernatant was used for PCR analysis. Primers for *clbQ* were used to identify *pks* genes^26^. Primers for *cnf* were used that amplify both *cnf1* and *cnf2*. Primers for all known variants of *cdtB* were used to detect *cdt*. Primers for *hlyA* were used to identify hemolysin genes. To determine the phylogenetic groups of isolates, multiplex PCR analysis was conducted using primers for *svg*, *chuA*, *yjaA*, *uidA*, and TspE4.C2. Amplicons produced distinct banding patterns with gel electrophoresis, allowing for differentiation of phylogroups A, B1, B2, and D^36,37,48^. The primers and annealing temperatures used are shown in Supplementary Table S4. Gels were stained with ethidium bromide and imaged using Syngene GeneSnap software. Gel images were edited for clarity. Unedited images of gels are shown in Supplementary Figure S6. Sanger sequencing (Quintara Biosciences, Cambridge, MA) of amplicons from select isolates was used to confirm sequence identity and to compare wildtype and mutant *hlyA* sequences.

### Clinical associations

Medical records were evaluated for temporal correlations from animals which had gastrointestinal clinical signs with culture of cyclomodulin-encoding *E. coli* isolates. Animals with more than three consecutive days of diarrhea were treated with oral enrofloxacin (5 mg/kg once daily for 5 days) and fecal samples were tested for GI pathogens. A presumptive diagnosis of IBD-like disease was made when an adult animal had bodyweight below 325 g and hypoalbuminemia below 3.5 g/dL with no identified infectious cause^49^. These animals were maintained on oral budesonide (0.5–0.75 mg once daily) to manage clinical signs. In animals with repeated bouts of vomiting and inappetence coupled with weight loss, a presumptive diagnosis of duodenal ulceration was made by palpation of a cranial abdominal mass, and often confirmed with abdominal ultrasound^50^. Many elements of this syndrome are similar to the duodenal dilation syndrome recently described^51^. These animals were maintained on oral sucralfate (100–200 mg/kg once daily) to manage clinical signs.

Animals with *E. coli* isolates collected in 2016 and 2017 were evaluated for stability of colonization by cyclomodulin-encoding *E. coli* over time. Animal records were also evaluated for associations of familial relationships with colonization by similar *E. coli* isolates based on biochemical analyses, phylogenetic groups, and virulence factor genotypes.

### Cytotoxicity assays

*Escherichia coli* strains used as controls in the cytotoxicity assays included K12 (*pks*-/*cnf*-/*cdt*-), V27 (a *pks*+/*cnf*−/*cdt*+ control from the *E. coli* Reference Center at Penn State), NC101 (a *pks*+/*cnf*−/*cdt*− mouse isolate generously gifted from Dr. Christian Jobin), and 1701240014 (a *pks*−/*cnf*+/*cdt*− rat isolate). Nine *E. coli* isolates from clinically normal marmosets were selected for more detailed analysis, and designated S1 through S9. These isolates represented all three marmoset colonies and all cyclomodulin genotype combinations present, including *pks*+/*cnf*+/*cdt*−, *pks*+/*cnf*−/*cdt*+, *pks*+/*cnf*−/*cdt*−, and *pks*−/*cnf*−/*cdt*−. These isolates were submitted for serotyping, and five of these isolates were selected for whole genome sequencing. HeLa S3 cells (CCL2.2, ATCC, Manassas, VA) were cultured in Eagle minimal essential medium (EMEM, ATCC) containing 10% FCS (Sigma, St Louis, MO) and 1% antibiotic–antimycotic (Gibco, Gaithersburg, MD) at 37 °C with 5% CO_2_. For both assays, 5,000 cells per well were seeded onto 96-well cell culture plates and incubated for 24 h (doubling time). Cells were then treated as described in the following sections. At the end of experiments, plates were stained with Diff-quick (Thermo Fisher Scientific, Waltham, MA). Cells were inspected for confluence and morphologic changes. Images were captured at 20× magnification with a Zeiss Axiovert-10 microscope (Jena, Germany) using Image Pro-Plus software version 7.0 (Media Cybernetics, Rockville, MD).

### Cell culture assay for colibactin cytotoxicity

The assay was performed as described previously with modifications^11,27^. Isolates of *E. coli* cultured overnight were incubated in LB broth for 2 h at 37 °C to reach logarithmic growth phase, then adjusted using OD600nm in 1% FCS EMEM to concentrations corresponding to multiplicities of infection (MOI) of 100, 25 and 5 bacteria per cell. After inoculation into 24-h non-confluent HeLa cultures, the 96-well plates were centrifuged at 200 g for 10 min to facilitate bacterial interaction, then incubated at 37 °C with 5% CO_2_ for 4 h. Cells were washed with EMEM and then incubated in EMEM containing 10% FCS and 200 µg/mL gentamicin (Gibco) at 37 °C with 5% CO_2_ for 72 h.

### Cell culture assay for sonicate cytotoxicity

Overnight cultures of *E. coli* isolates were suspended in PBS and pelleted by centrifugation at 12,000 rpm for 10 min at 4 °C. The pellets were resuspended in 1.5 mL of PBS and sonicated on ice (amplitude: 35, power: 7 W) for a total of 5 min divided into 30-s intervals, with 60-s breaks between intervals to prevent overheating. Sonicated samples were centrifuged at 12,000 rpm for 10 min at 4 °C. Supernatants were collected and filter-sterilized through 0.2 µm filters. Total proteins were quantified using the BCA assay (Thermo Fisher Scientific), then 24-h non-confluent HeLa cultures were treated with crude bacterial sonicate (80, 140, or 220 µg/mL total protein) or PBS (40 µL) and incubated at 37 °C with 5% CO_2_ for 72 h.

### Serotyping

The nine representative *E. coli* isolates, S1–S9, were submitted to the *E. coli* Reference Center at The Pennsylvania State University for serotype testing, which included O and H typing as well as PCR analyses for heat-labile enterotoxin (*elt*), heat stable enterotoxins A and B (*estA* and *estB*), Shiga toxins 1 and 2 (*stx1* and *stx2*), cytotoxic necrotizing factor 1 and 2 (*cnf1* and *cnf2*), and intimin gamma (*eae*).

### Draft genome sequencing and comparative analysis

Genomic DNA was isolated using the High Pure PCR Template Preparation Kit (Roche Molecular Biochemicals, Indianapolis, IN) following the manufacturer’s protocol for bacterial cell samples. DNA libraries were prepared using the QIAseq FX DNA Library Kit (Qiagen, Valencia, CA) following the manufacturer’s protocol for 500 bp fragments. DNA libraries were sequenced using 2 × 300 bp paired-end reads by Illumina MiSeq by the MIT BioMicro Center. Raw sequenced reads were decontaminated of adapter sequences and quality trimmed to a Phred quality score (Q) ≥ 10 using BBDuk from the BBMap package version 38.34 (http://sourceforge.net/projects/bbmap/). Decontaminated reads were then assembled into contigs with SPAdes followed by genome annotation with RAST, both services hosted by PATRIC^52^ .Sequences encoding putative virulence factor and antibiotic-resistance genes were identified using PathogenFinder 1.1^53^, VirulenceFinder 2.0^54^, and ResFinder 3.2^55^ using the 90% identity and 60% minimum length threshold parameters, as described previously^26^. Syntenic relationships of *pks* genes and the *hlyCABD*-*cnf1* operon between genomes were determined with SimpleSynteny v1.4^56^, as described previously^26^. The “Similar Genome Finder” tool, hosted by PATRIC^52^, was used to identify *E. coli* genomes that were related to the marmoset *E. coli* isolate genomes. Sequences have been deposited in GenBank under the following accession numbers JAADCB000000000, JAADBZ000000000, JAADCA000000000, JAADCC000000000, and JAADBY000000000 for isolates S1, S3, S4, S5, and S8, respectively (Table 2).

## Statistical analysis

A webtool was used to calculate expected contingency tables and to perform 2-tailed Fisher exact tests to evaluate categorical data with small group sizes. Differences were determined between categories of virulence factor, phylogroup, and API code, among the three marmoset colonies and taking into account marmoset age, sex and health status^57^. Statistical significance was set at a *P* value of less than 0.05.

### Data availability

The datasets generated during and/or analyzed during the current study are available from the corresponding author on reasonable request.

## Supplementary Information

Supplementary Information 1.
