# Supplementary material for: Cytotoxic Escherichia coli strains encoding colibactin, cytotoxic necrotizing factor, and cytolethal distending toxin colonize laboratory common marmosets (Callithrix jacchus)
Source: Sci Rep. 2021 Jan 27;11:2309. doi: 10.1038/s41598-020-80000-1 (PMC7841143; doi:10.1038/s41598-020-80000-1)
Supplement: Supplementary file 1 — Supplementary Information 1. [file 41598_2020_80000_MOESM1_ESM.pdf]

Cytotoxic *Escherichia coli* strains encoding colibactin, cytotoxic necrotizing factor, and cytolethal distending toxin colonize laboratory common marmosets (*Callithrix jacchus*)

Colleen S. McCoy[1], Anthony J. Mannion[1], Yan Feng[1], Carolyn M. Madden[1], Stephen C. Artim[1], Gina G. Au[1], Mikayla Dolan[1], Jennifer L. Haupt[1], Monika A. Burns[1], Alexander Sheh[1], and James G. Fox[1*]

[1]Division of Comparative Medicine, Massachusetts Institute of Technology, Building 16-825, 77 Massachusetts Avenue, Cambridge, Massachusetts, 02139, USA
Stephen C. Artim's current address: Merck Research Laboratories, Merck, South San Francisco, CA 94080, USA
[*]jgfox@mit.edu

Supplementary Information

Contents	Description	Page
Table S1	*E coli* isolates from sampled marmosets by colony	2
Figure S1	Biochemical characteristics of *E. coli* isolates in each marmoset colony	2
Table S2	Distribution of cyclomodulin prevalence in *E. coli* isolates in each marmoset colony	3
Figure S2	Evidence of stable colonization of marmosets by *pks*+ *E. coli*	4
Figure S3	Demonstration of the presence of mutant hemolysin and its distribution in marmoset colonies	5
Figure S4	Cytotoxicity assays of representative *E. coli* isolates	6
Figure S5	Syntenic gene alignments for *pks* and *hlyCABD–cnf1* operon	7
Table S3	Similar genomes for selected *E. coli* isolates	8
Table S4	Genes, primers, and annealing temperature used for PCR amplification	9
Figure S6	Unedited images of PCR gels	10
	Supplementary References	11

Table S1. *E coli* isolates from each marmoset colony

	Total	Colony A	Colony B	Colony C
Animals positive for *E. coli*	122/142 (86%)	26/33 (79%)	32/33 (97%)	64/77 (83%)
Number of *E. coli* isolates	139	31	33	75

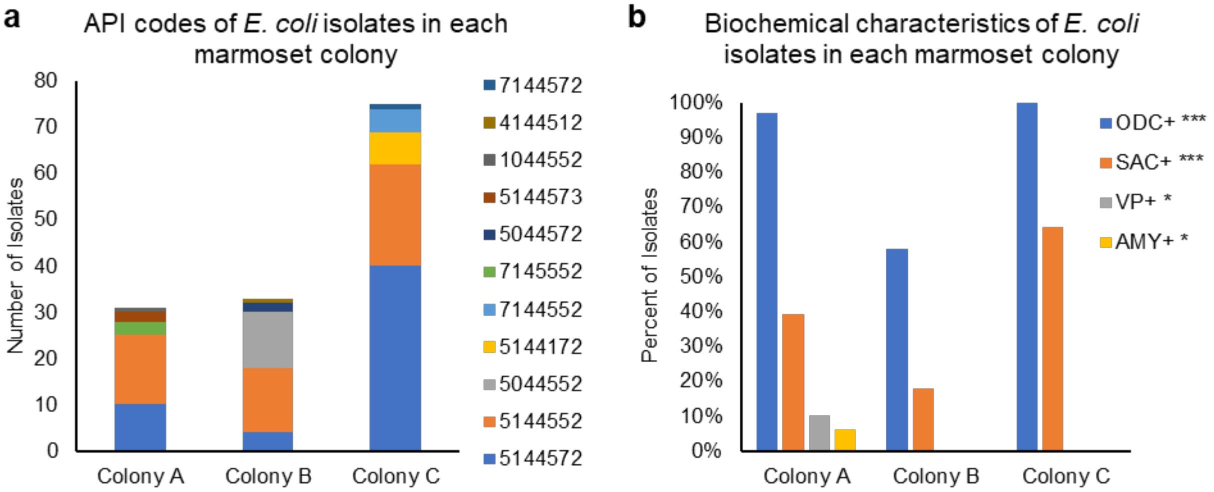

Figure S1. Biochemical characteristics of *E. coli* isolates in each marmoset colony. (**a**) Of the 139 total *E. coli* isolates, 54 were API code 5144572, and 51 isolates were API code 5144552, constituting the majority of isolates present in all three marmoset colonies. (**b**) There were significant correlations of marmoset colony of origin with different biochemical characteristics, including the presence of ornithine decarboxylase (ODC), sucrose fermentation (SAC), butylene glycol pathway (VP), and amygdalin fermentation (AMY). Fisher's Exact Test. * $p<0.05$, *** $p<0.001$

Table S2. Distribution of cyclomodulin prevalence in *E. coli* isolates in each marmoset colony.

Genotype	Total	Colony A	Colony B	Colony C
Total *pks*+ *E. coli*	56/139 (40%)	18/31 (58%)	2/33 (6%)***	36/75 (48%)
Total *cnf*+ *E. coli*	47/139 (34%)	17/31 (55%)	0/33 (0%)***	30/75 (40%)
Total *cdt*+ *E. coli*	1/139 (0.7%)	1/31 (3%)	0/33 (0%)	0/75 (0%)
pks+/*cnf*+/cdt-	47	17	0	30
pks+/cnf-/cdt+	1	1	0	0
pks+/*cnf*-/cdt-	8	0	2	6
pks-/*cnf*-/cdt-	83	13	31	39

*** Colony B had significantly fewer *pks*+ and *cnf*+ *E. coli* isolates than Colony A and Colony C. (Fisher's Exact Test, $p<0.001$)

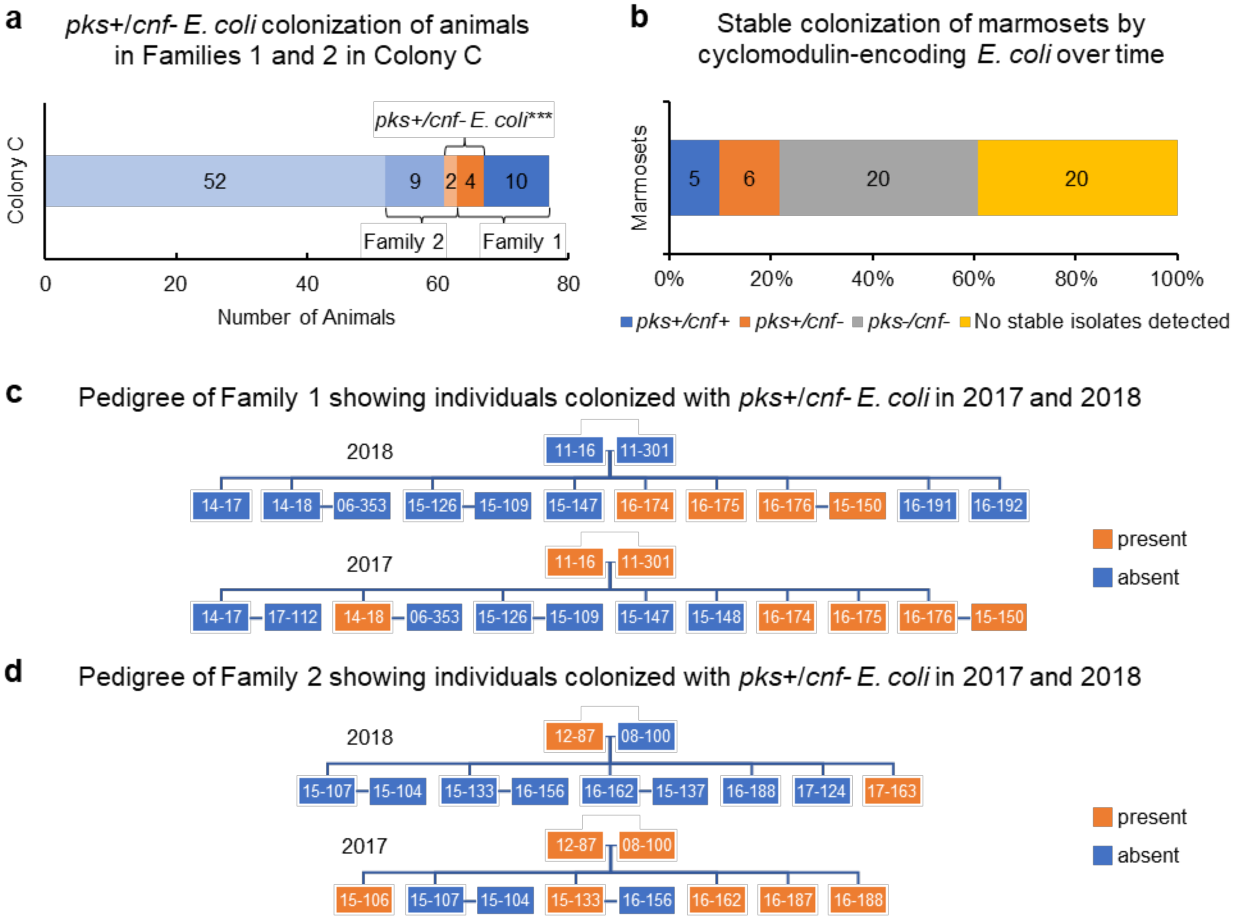

Figure S2. Evidence of stable colonization of marmosets by *pks*+ *E. coli*. (**a**) Animals colonized by *pks*+/*cnf*- *E. coli* in 2018 were found within the 2 largest extended families in Colony C, and had significantly different prevalence in these two families (24%) from the prevalence in the rest of Colony C (0%). *** $p < 0.001$, Fisher's Exact Test (**b**) Proportion of all marmosets sampled multiple years that had stable colonization by cyclomodulin-encoding *E. coli*. The number of animals within each category is written on the corresponding section of the bar graph. (**c** and **d**) Pedigrees of Family 1 (**c**) and Family 2 (**d**), the largest extended families in Colony C, showing individuals that were colonized by *pks*+/*cnf*- E. coli in 2017 and 2018 in orange. In each pedigree, parents are represented on the top tier, connected by vertical lines to offspring on the second tier, who are connected by horizontal lines to their unrelated cohoused mates. (Note: Individuals 16-191, 16-192, 17-124, and 17-163 were not tested in 2017 due to their age; 17-112 and 15-148 were relocated to another institution before the 2018 test; 15-106 and 16-187 were deceased before the 2018 test).

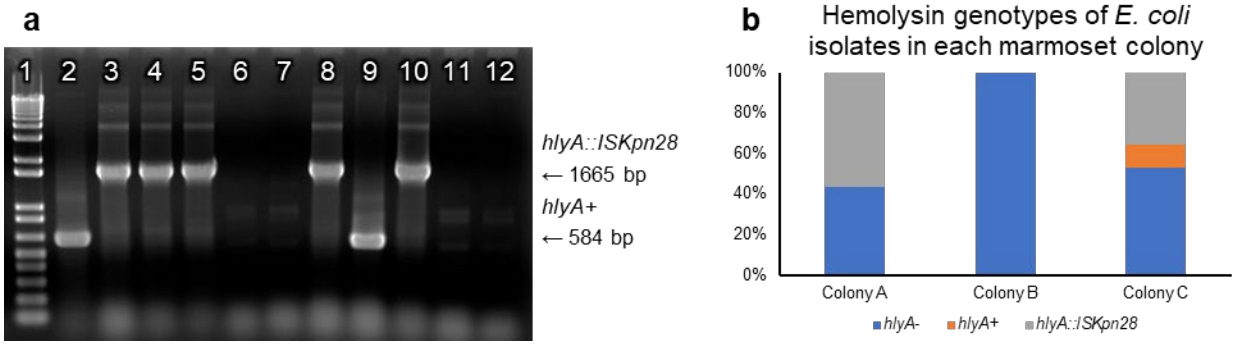

Figure S3. Demonstration of the presence of mutant hemolysin and its distribution in marmoset colonies. (**a**) Insertional mutations were frequently detected in the hemolysin gene (*hlyA*) of *cnf*+ *E. coli* isolates that did not demonstrate hemolytic activity on blood agar plates. Expected *hlyA* PCR product size is 584 bp. Hemolysin genes with insertional mutation produced a band at 1665 bp. PCR gels: lane 1,1-kb ladder; lane 2, positive control; lanes 3-11, representative E. coli isolates from marmosets; lane 12, negative control. (**b**) Distribution of wild type (*hlyA*) and mutant hemolysin genes by colony. All three marmoset colonies had significantly different prevalence of wildtype and mutant hemolysin genes (Fisher's Exact Test, $p < 0.001$). *hlyA*-, hemolysin gene not present; *hlyA*+, wild-type hemolysin gene present; *hlyA::ISKpn28*, mutant hemolysin gene with insertional element present. Unedited images of gels are shown in Supplementary Figure S6.

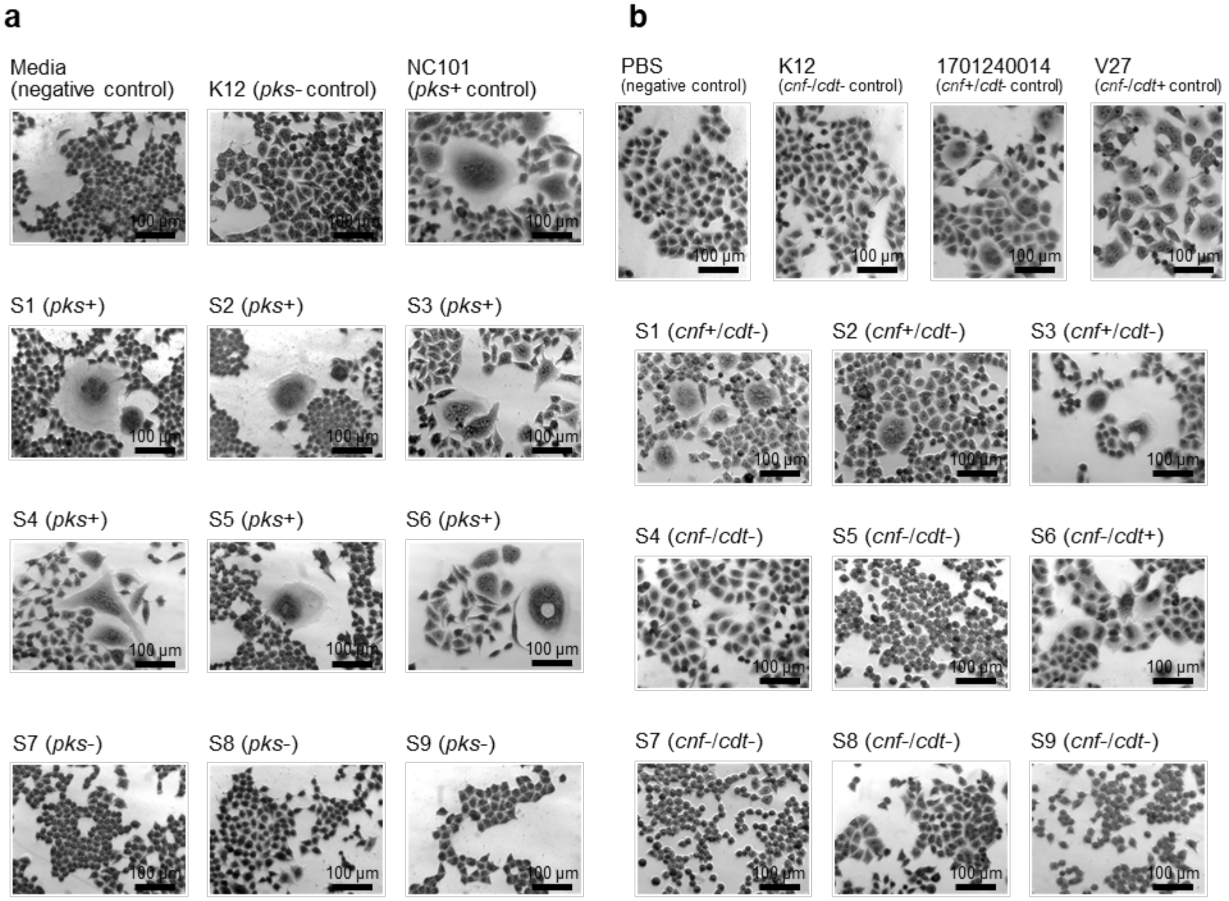

Figure S4. Cytotoxicity assays of representative *E. coli* isolates. (**a**) Cell culture assay to detect colibactin cytotoxicity using HeLa cells infected with live *E. coli* at multiplicity of infection (MOI) of 25. (**b**) Cell culture assay to determine CNF cytotoxicity using HeLa cells treated with sonicate of *E. coli* isolates at a dose of 140 μg/ml total protein.

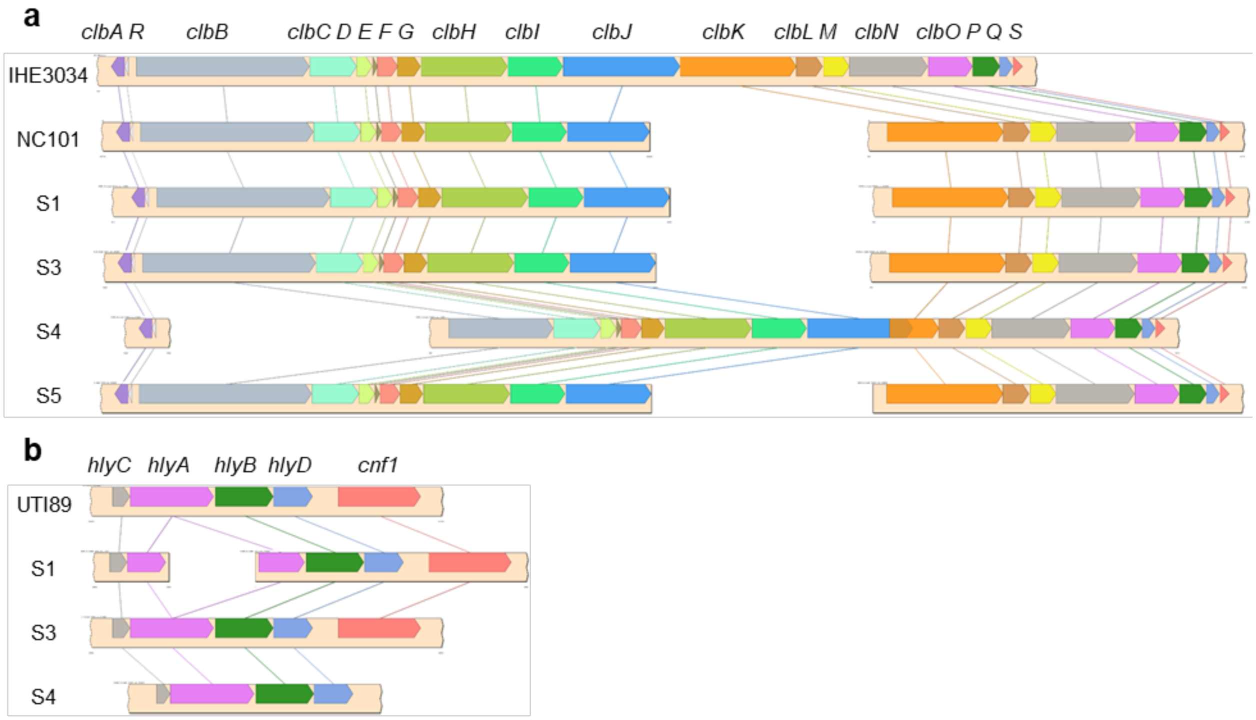

Figure S5. Syntenic gene alignments, shown to relative scale of gene length (**a**) *pks* island genes of IHE3034, NC101, and four representative novel marmoset *E. coli* isolates. S4 harbors a hybrid *clbJ-K* sequence. (**b**) *hlyCABD–cnf1* operon syntenic alignment between *cnf*+ UTI89 uropathogenic *E. coli* and three representative novel marmoset *E. coli* isolates. The S4 isolate was PCR positive for *hlyA*, but not for *cnf*, which corresponds to the lack of BLAST alignment to *cnf1* depicted here. A 1081-bp insertion is present in the *hylA* gene of S1, which rendered it nonfunctional.

Table S3. Similar Genome Finder top five matches for *E. coli* strains S1, S3, S4, S5 and S8. Marmoset *E. coli* strains had significantly similar genomes to human and animal EPEC and UPEC strains found worldwide.

Strain	Similar Genome	GenBank Accession	Sequencing Center	Source	Location	Date Collected	Comments	Distance	P Value	Counts
S1	*E. coli* strain EEITKB331	SQCD01000000	University of Tartu	Human	Estonia	2012	Investigation of antibiotic resistance	0.00266	0	897/1000
S1	*E. coli* strain 582A	UIMH01000000	Hopital Henri Mondor APHP	Human Rectal Swab	France	2018	Prevalence of colistin resistant *E. coli* in humans	0.00280	0	892/1000
S1	*E. coli*	CABWIP010000001-CABWIP010000007	UMR 1137 IAME	Human Feces	Sweden	2000	Investigation of ExPEC and EPEC in healthy infants	0.00286	0	890/1000
S1	*E. coli* strain Aut2013_WWKa_OUT_20	NBKS01000000	BIOTEC	Wastewater Outflow	Dresden-Kaditz, Germany	2013	Samples of *E. coli* from inflow and outflow of a wastewater treatment plant	0.00294	0	887/1000
S1	*E. coli* strain EETUKB165	SQMR01000000	University of Tartu	Human	Estonia	2012	Investigation of antibiotic resistance	0.00329	0	875/1000
S3	*E. coli* strain MOD1-EC5193	NLRA00000000	FDA/CFSAN	Canine Urine	Florida, USA	1985	FDA surveillance for the rapid detection of foodborne contamination events	0.00149	0	940/1000
S3	*E. coli* strain 11_ECOL	JWCY01000000	University of Washington	Human	Washington, USA	---	Bacterial isolate from a hospital's intensive care units	0.00165	0	934/1000
S3	*E. coli* strain MOD1-EC709	NJKD01000000	FDA/CFSAN	Human Blood	Washington, USA	1985	FDA surveillance for the rapid detection of foodborne contamination events	0.00165	0	934/1000
S3	*E. coli* strain 20.1a	NIDY00000000	Broad Institute	Human Urine	Seattle, Washington, USA	2007	Clinical Sample: Urinary Tract Infection	0.00167	0	933/1000
S3	*E. coli* UMEA 3290-1	AYHJ00000000	Broad Institute	Human Urine	Sweden	1995	Clinical Sample: Urinary Tract Infection; Bacteremia	0.00167	0	933/1000
S4	*E. coli* O6:H31 strain ECO0896	---	---	Human	UK	---	No information given	0.00210	0	917/1000
S4	*E. coli* strain K56-16	PIJJ01000000	The Arctic University of Norway	Human Urine	Portugal	2000	Clinical Sample: Urinary Tract Infection	0.00216	0	915/1000
S4	*E. coli* UMEA 3022-1	AWAU00000000	Broad Institute	Human Urine	Sweden	1995	Clinical Sample: Urinary Tract Infection; Bacteremia	0.00221	0	913/1000
S4	*E. coli* UMEA 3341-1	AWDJ00000000	Broad Institute	Human Urine	Sweden	1996	Clinical Sample: Urinary Tract Infection; Bacteremia	0.00224	0	912/1000
S4	*E. coli* UMEA 3217-1	AWCP00000000	Broad Institute	Human Urine	Sweden	1995	Clinical Sample: Urinary Tract Infection; Bacteremia	0.00227	0	911/1000
S5	*E. coli* strain 40_rectal	SCJC01000000	Washington University	Human Rectal Swab	St. Louis, Missouri, USA	2013	Study on Catheter Associated UTI: Healthy Control Sample	0.00213	0	916/1000
S5	*E. coli* strain GN03545	LQVY01000000	JCVI	Human	USA	2010	Study on Evolution of *E. coli* in Bacteremia	0.00232	0	909/1000
S5	*E. coli* strain MOD1-EC6692	NOUK01000000	FDA/CFSAN	Human Blood	California, USA	1992	FDA surveillance for the rapid detection of foodborne contamination events	0.00235	0	908/1000
S5	*E. coli* upec-60	JSHD00000000	University of Washington	Human Urine	Washington, USA		Clinical Sample: Urinary Tract Infection	0.00238	0	907/1000
S5	*E. coli* upec-156	JSMU00000000	University of Washington	Human Urine	Washington, USA	2011	Clinical Sample: Urinary Tract Infection	0.00238	0	907/1000
S8	*E. coli* strain Sum2013_WWKa_OUT_31	NBDC01000000	BIOTEC	Wastewater Outflow	Dresden-Kaditz, Germany	2013	Samples of *E. coli* from inflow and outflow of a wastewater treatment plant	0.00191	0	924/1000
S8	*E. coli* strain MOD1-EC6363	NMIN01000000	FDA/CFSAN	Psittacine Feces	Texas, USA	1987	FDA surveillance for the rapid detection of foodborne contamination events	0.00251	0	902/1000
S8	*E. coli* strain C60	NDBB00000000	University of Sao Paulo	Crassostrea Oyster	Santos, Brazil	2016	Extended-spectrum-beta-lactamase-producing *E. coli* isolated from seafood	0.00257	0	900/1000
S8	*E. coli* strain MPEC4969	JYHQ00000000	Georg-August-University Goettingen	Bovine Udder	Israel	2009	Comparative genomics of *E. coli* bovine mastitis and fecal commensal isolates	0.00260	0	899/1000
S8	*E. coli* strain 208_3	QERP01000000	Universidad de Chile	Bovine Feces	Santiago, Chile	2003	Characterization of *E. coli* isolates	0.00265	0	897/1000

Table S4. Genes, primers, and annealing temperature used for PCR amplification.

**Gene**	**Primer Designation**	**Primer Sequence (5' to 3')**	**Annealing Temp (°C)**	**Ref**
clbQ	IHAPJPN55 (clbQr)	TTATCCTGTTAGCTTTCGTTC	55	1
	IHAPJPN56 (clbQf)	CTTGTATAGTTACACAACTATTTC		
multiplex *cnf*	CNF-s	TTATATAGACAAGATGGA	55	2
	CNF-as	CATAAAGCTTTACAATATTGA		
multiplex *cdt*	CDT-s1	GAAAGTAAATGGAATATAAATGTCCG	55	2
	CDT-as1	AAATCACCAAGAATCATCCAGTTA		
hlyA	hem1a	TTGCTGAATGGGAGAAAAA	51	3
	hem1b	ACCTGCGTAGATATTGGCTGAG		
yjaA	YjaA.1	TGAAGTGTCAGGAGACGCTG	55	4
	YjaA.2	ATGGAGAATGCGTTCCTCAAC		
TSPE4.C2 (DNA fragment)	TspE4C2.1	GAGTAATGTCGGGGCATTCA	55	4
	TspE4C2.2	CGCGCCAACAAAGTATTACG		
chuA	ChuA.1	GACGAACCAACGGTCAGGAT	55	4
	ChuA.2	TGCCGCCAGTACCAAAGACA		
svg	svg.1	TCCGGCTGATTACAAACCAAC	55	5
	svg.2	CTGCACGAGGTTGTAGTCCTG		
uidA	P3	TATGAACTGTGCGTCACAGCC	55	6
	P4	CATCAGCACGTTATCGAATCC		

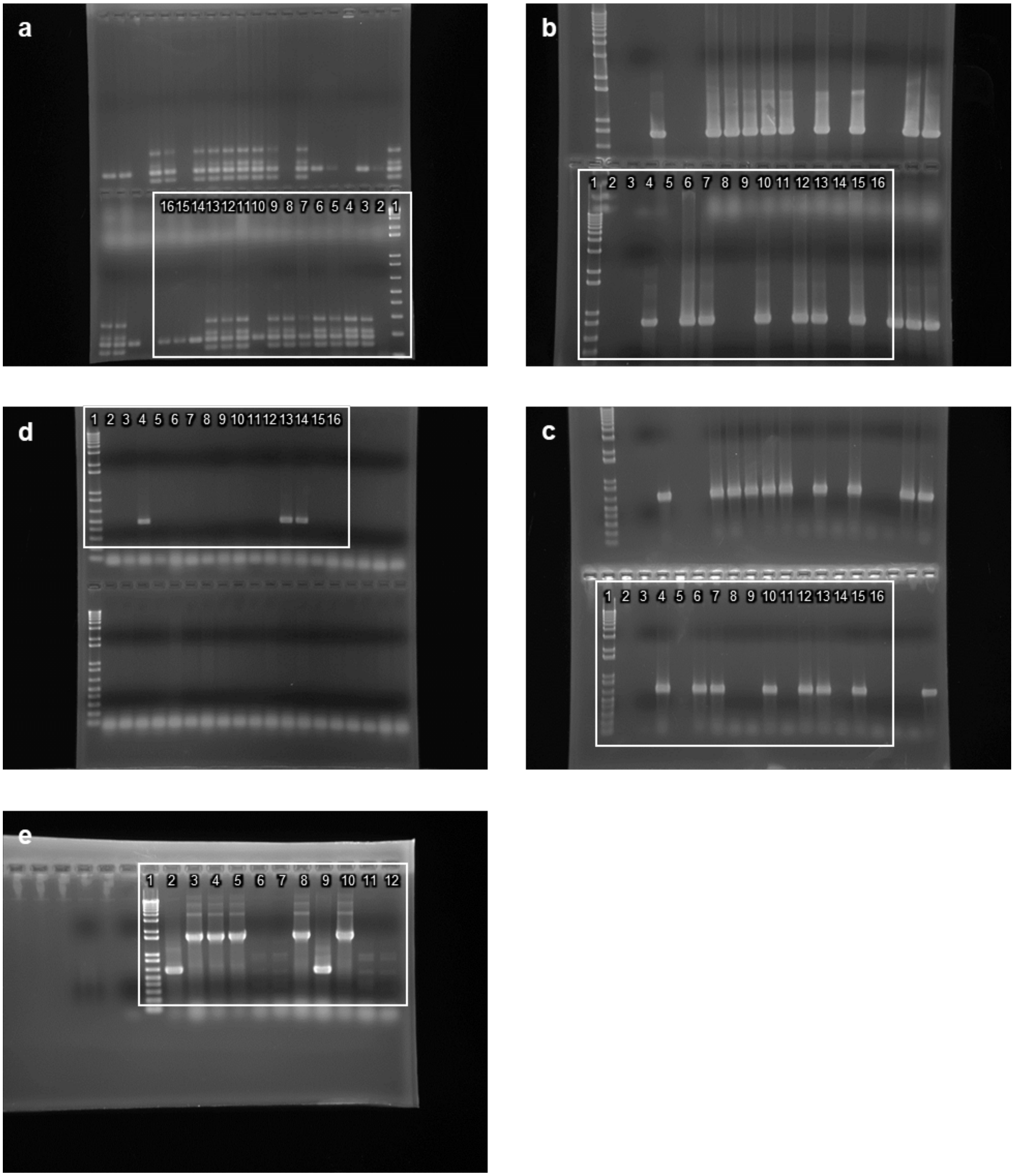

Figure S6. Full size images of PCR gels depicted in Figures 1 and 2, and Supplementary Figure S3. Areas highlighted in main figures are outlined in white and lanes of interest are labeled accordingly. (**a**) Phylogroup multiplex PCR gel: lane 1,1-kb ladder; lane 2, negative control; lane 3, phylogroup B2 positive control (NC101); lanes 4-16, representative *E. coli* isolates from marmosets (lanes 3-9 and 11-13, phylogroup B2; lanes 10 and 14-16, phylogroup B1). (**b**) *pks*, (**c**) *cnf*, and (**d**) *cdt* PCR gels: lane

1,1-kb ladder; lane 2, blank; lane 3, negative control; lane 4, positive control; lanes 5 through 16, representative *E. coli* isolates from marmosets. (**e**) *hlyA* and *hlyA::ISKpn28* PCR gels: lane 1,1-kb ladder; lane 2, positive control; lanes 3-11, representative *E. coli* isolates from marmosets; lane 12, negative control.

Supplementary References

1. Nougayrède, J.P., et al. *Escherichia coli* induces DNA double-strand breaks in eukaryotic cells. *Science* **313**, 848-851, https://doi.org/10.1126/science.1127059 (2006).
2. Tóth, I., Hérault, F., Beutin, L. & Oswald, E. Production of cytolethal distending toxins by pathogenic *Escherichia coli* strains isolated from human and animal sources: establishment of the existence of a new *cdt* variant (Type IV). *J. Clin. Microbiol*. **41**, 4285-4291, https://doi.org/10.1128/jcm.41.9.4285-4291.2003 (2003).
3. Kurnick, S.A., et al. Genotoxic *Escherichia coli* strains encoding colibactin, cytolethal distending toxin, and cytotoxic necrotizing factor in laboratory rats. *Comp. Med*. **69**, 103-113, https://doi.org/10.30802/AALAS-CM-18-000099 (2019).
4. Clermont, O., Bonacorsi, S. & Bingen, E. Rapid and simple determination of the *Escherichia coli* phylogenetic group. *Appl. Environ. Microbiol*. **66**, 4555-4558, https://doi.org/10.1128/aem.66.10.4555-4558.2000 (2000).
5. Bidet, P., et al. Detection and identification by PCR of a highly virulent phylogenetic subgroup among extraintestinal pathogenic *Escherichia coli* B2 strains. *Appl. Environ. Microbiol*. **73**, 2373-2377, https://doi.org/10.1128/AEM.02341-06 (2007).
6. Heininger, A., et al. PCR and blood culture for detection of *Escherichia coli* bacteremia in rats. *J. Clin. Microbiol*. **37**, 2479-2482, (1999).
